# Plasmon-Enhanced Surface Photovoltage of ZnO/Ag Nanogratings

**DOI:** 10.1038/srep16727

**Published:** 2015-11-16

**Authors:** Minji Gwon, Ahrum Sohn, Yunae Cho, Soo-Hyon Phark, Jieun Ko, Youn Sang Kim, Dong-Wook Kim

**Affiliations:** 1 Department of Physics, Ewha Womans University, Seoul 120750, Korea; 2 Center for Correlated Electron Systems, Institute for Basic Science (IBS), Seoul 151-747, Korea; 3 Department of Physics and Astronomy, Seoul National University, Seoul 151-742, Korea; 4 Program in Nano Science and Technology, Graduate School of Convergence Science and Technology, Seoul National University, Seoul 151-742, Korea; 5 Advanced Institutes of Convergence Technology, Suwon, Gyeonggi-do 443-270, Korea

## Abstract

We investigated the surface photovoltage (SPV) behaviors of ZnO/Ag one-dimensional (1D) nanogratings using Kelvin probe force microscopy (KPFM). The grating structure could couple surface plasmon polaritons (SPPs) with photons, giving rise to strong light confinement at the ZnO/Ag interface. The larger field produced more photo-excited carriers and increased the SPV. SPP excitation influenced the spatial distribution of the photo-excited carriers and their recombination processes. As a result, the SPV relaxation time clearly depended on the wavelength and polarization of the incident light. All of these results suggested that SPV measurement using KPFM should be very useful for studying the plasmonic effects in nanoscale metal/semiconductor hybrid structures.

ZnO, a wide-bandgap semiconductor, is a strong candidate material for many applications, including transparent conducting electrodes^1^, photodetectors^2^,^3^,^4^, UV LEDs^5^, field-effect transistors^6^,^7^,^8^, sensors^9^, and energy harvesting devices^10^. Optical characterizations of metal-ZnO hybrid nanostructures have revealed interactions between SPs and charge carriers^1^,^11^,^12^,^13^,^14^,^15^. Such plasmonic effects can also be investigated by electrical measurements^16^, but elaborate fabrication processes are often required. Near-field scanning optical microscopy (NSOM) and electron-energy-loss spectroscopy (EELS) allow us to study the SP excitation behaviors of metal nanostructures^17^,^18^. NSOM can visualize subwavelength-scale concentrated EM fields and EELS can show the amount of energy loss of incoming electrons with tens-of-nanometers spatial resolution. Neither of these tools, however, can directly reveal how the carriers in the optical active materials, placed adjacent to the metal nanostructures, interact with SPs.

Kelvin probe force microscopy (KPFM), a variant of atomic force microscopy (AFM), is a technique used to measure the local surface potential of a sample^19^,^20^,^21^,^22^,^23^. KPFM has the advantage of being a non-destructive ambient-operation tool; it has consequently been successfully used to measure the local doping concentration^20^, the band bending near structural defects^21^, and the domain configuration in a sample containing two distinct phases^22^. KPFM also enables us to obtain spatial maps of surface photovoltage (SPV), i.e., changes in the surface potential under illumination. SPV originates from the photo-excitation of carriers and subsequent redistribution of net charges^23^,^24^. The aforementioned SP-charge coupling should play key roles in the creation and recombination of the charge carriers. Thus, KPFM experiments would help elucidate the interaction between the SPs excited by incoming photons and the charge carriers in the active materials.

We investigated the SPV behaviors of ZnO/Ag nanogratings using KPFM under illumination by linearly polarized light. The grating structure can couple surface plasmon polaritons (SPPs) at the ZnO/Ag interface with incident photons. The KPFM measurements revealed that the SPP excitation influenced the amount and relaxation time of SPV. We demonstrate that KPFM measurement can reveal the interaction between SPs and charge carriers in nanoscale metal/semiconductor hybrid structures.

One-dimensional (1D) ZnO/Ag nanograting structures, with a period of 1 μm and a line-to-space ratio of 1:1, were prepared on polymer patterns, fabricated by imprint lithography, as shown in Fig. 1a–c. 100-nm-thick ZnO and 100-nm-thick Ag thin films were deposited on the nanopatterns at room temperature. For comparison, ZnO/Ag thin films were also prepared on flat Si wafers. Details of the sample fabrication procedures are available elsewhere^14^ and the Methods section. Because of the limited step coverage, the thickness of the both ZnO and Ag thin films at the sidewalls of the nanograting was only 38 nm, as shown in the scanning electron microscopy image (Fig. 1b) and the schematic of the nanograting (Fig. 1c). Figure 1d shows a schematic of our KPFM measurement system (XE-100, Park Systems) in a glove box filled with N_2_ gas. We used 5 mW green and red laser modules with wavelengths of 532 nm and 635 nm (LDC series, Korea), respectively, for the SPV measurements. The light was aligned to illuminate the sample area under the KPFM tip with an incident angle of 65°. At smaller angles, part of the laser light was blocked by the KPFM head. Depending on the incident angle, the energy of light-coupled SPP mode can be determined by the SPP dispersion relation (see Figure S1). The polarization of the laser light was adjusted with a linear polarizer to either transverse electric (TE) or transverse magnetic (TM) mode. Prior to the work-function measurements, the samples were annealed at 100 °C for 30 min to remove adsorbed water^22^,^23^.

Figure 2a–f show topography and work-function (*W*_*S*_) maps of the ZnO/Ag nanograting. All of the images were obtained at the top flat region of the grating, because the tip-sample convolution did not allow reliable measurements at the lower part of the grating^19^,^20^. As shown in Fig. 2a,d, the sample surface has mounds with sizes on the order of tens of nanometers; these mounds are presumed to be grains formed during growth and/or heat treatment before the KPFM measurements. The dark-state *W*_*S*_ values are similar to those reported in the literature^19^ and show spatial fluctuation (Fig. 2b,e). Similar electronic inhomogeneity has also been reported in other KPFM studies of ZnO thin films^19^,^20^. Doping concentrations and oxygen adsorbates can alter the *W*_*S*_ of the ZnO thin films^19^,^20^,^23^. Jaramillo and Ramanathan reported that larger (smaller) *W*_*S*_ appeared at the grain boundaries (over the grains) of optimally oxidized ZnO thin films^19^. A comparison of the topographic images (Fig. 2a,d) and *W*_*S*_ maps in the dark (Fig. 2b,e) reveals no clear relationship in the spatial distributions of the grain boundaries and regions with large (or small) *W*_*S*_, similar to the results of Maragliano *et al.*^20^. This result suggests that our ZnO thin films could be insufficiently oxidized^20^. The oxidation state of our samples should be determined by the growth conditions and heat treatment prior to the KPFM measurements.

Figure 2c,f show that overall *W*_*S*_ values under illumination of green light (wavelength: 532 nm) are somewhat smaller than those in dark (i.e., the area of blue-colored region increases). SPV is defined as difference between *W*_*S*_ in dark (*W*_*dark*_) and *W*_*S*_ in light (*W*_*light*_), i.e., SPV = *W*_*dark*_ − *W*_*light*_, and hence SPV has positive sign in both TM and TE mode green light illumination. It should be noted that the TM mode light induces much larger SPV than the TE mode. Spatial distributions of *W*_*dark*_ and *W*_*light*_ do not show any notable correlation to each other. The *W*_*light*_ maps exhibit no similarity to the topographic images, like the *W*_*dark*_ maps. This suggests that the topographic artifact hardly affects the work function measurements of our samples.

As shown in Fig. 3 (green arrows), illumination with sub-bandgap-energy light can generate photo-excited electrons and holes via the trap-to-band transitions^24^. (Note: the photon energy of the green light, 2.33 eV, is lower than the bandgap energy of ZnO, 3.35 eV.) Our ZnO thin films should have numerous defects both at the surface and in the bulk because they were grown at room temperature. As a result, the photoluminescence spectra of our ZnO/Ag nanogratings exhibited strong trap-state-mediated visible emission as well as the UV emission caused by the band-to-band excitation^14^,^15^. The visible emission from ZnO in the energy range of 1.6–2.4 eV has been attributed to interstitial Zn ions, Zn-vacancy-related defects, oxygen vacancies, and chemisorbed oxgens^13^,^25^. The surface of the ZnO sample may contain more defects than the interior, and the surface defects readily act as adsorption sites^2^,^4^. Gas molecules in air are readily chemisorbed onto the ZnO surface by the capture of free electrons, leading to the upward band bending at the surface, as depicted in Fig. 3. The resulting electric field repels (attracts) the photo-excited electrons (holes) from (to) the surface^24^. This redistribution of charge carriers reduces the band bending and decreases *W*_*S*_ (i.e., SPV = *W*_*dark*_ − *W*_*light*_ > 0), as observed in the experimental results shown in Fig. 2 25,26. If we have sufficiently large number of charge carriers, then the surface band bending can be nullified under illumination and no further electron-hole pair separation occurs. Thus, the measured SPV cannot be larger than the surface band bending of the sample. Large surface density can increase electron transitions with the same photon flux, and hence increases SPV. Theoretical studies showed that the sub-bandgap SPV could be much less than the surface band bending and largely varied depending on the surface and bulk density of states^24^.

Figure 4 shows the SPV values obtained from a ZnO/Ag thin film and the ZnO/Ag nanograting, when the red and green light was illuminated with TM- and TE-mode polarizations. The SPV value could be estimated at each pixel of the *W*_*S*_ maps (for the nanograting, see Fig. 2a–f). The average data and the statistical distributions are shown in Fig. 4. The SPV values of the thin film and the nanograting under the green light are larger than those under the red light. Such wavelength dependence is determined by the trap state energy distribution of ZnO thin films^25^. Under the green light with TM mode, the SPV of ZnO/Ag is the largest (~60 meV) among all of the measured SPV values. In contrast, the polarization dependence of the flat thin film is not very notable. Also, it can be noted that the SPV under the red light hardly depends on the polarization of the incoming light for both the thin film and nanograting samples. The excitation of SPP via light is not very efficient in flat samples, since the momentum of the SPP mode is greater than that of a free-space photon of the same frequency^14^. In the nanograting, such momentum mismatch can be overcome and SPP with the specific energy range from 2 eV to 3 eV can be excited in our ZnO/Ag nanograting samples (see Figure S1). Thus red light with a wavelength of 635 nm (1.95 eV) could not excite SPP, but green light with a wavelength of 532 nm (2.33 eV) could. The wavelength and polarization dependence of SPV may suggest that the interaction between SPPs at the ZnO/Ag interface and charge carriers in the ZnO layer could affect the SPV behaviors of our ZnO/Ag nanogratings.

Figure 5 shows the electric field intensity, 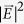, distributions in the ZnO/Ag nanograting, as obtained by finite-difference time-domain (FDTD) simulations (Lumerical FDTD Solutions)^14^,^15^. The light has an incident angle of 65°, as depicted in Fig. 1d: the electric (magnetic) field of the TE- (TM-) mode light is parallel to the 1D grating. The diffraction, scattering, and interference of light in the samples caused intensity modulation. A very strong field confined at the ZnO/Ag interface is shown only for the case of TM-mode green-light illumination, indicating SPP excitation (Fig. 5b). In contrast, the TE-mode light in the grating has a much smaller intensity near the ZnO/Ag interface. The planar ZnO/Ag thin film, where SPP cannot exist, has a relatively uniform field intensity in the whole ZnO layer compared with the nanograting sample. The simulation results also showed that illumination with the red light could not excite SPP, as expected (see Figure S2). The larger field intensity should generate more photo-excited carriers, resulting in larger SPV. This situation clearly explains why the nanograting under TM-mode green light illumination exhibits the largest SPV (Fig. 4).

After the laser light was turned off, the equilibrium charge distribution can be recovered and SPV exhibited relaxation, as depicted in Fig. 6. The relaxation time strongly depends not only on the sample type (grating vs. flat sample) but also on the polarization direction of the incident light. Long-lasting photocurrent decay, so-called persistent photoconductivity (PPC), has been reported in ZnO thin films and nanostructures^2^,^3^,^4^,^5^. The noticeable relaxation behaviors of SPV in our ZnO/Ag nanogratings should have common physical origins with the PPC phenomena. Under illumination, the aforementioned surface band bending promotes the spatial separation of photo-generated electrons and holes, thereby increasing the recombination lifetime of the carriers, *τ*. In addition, the surface-migrated holes facilitate desorption of the chemisorbed oxygen molecules at the surface (O_2_^−^_(ad)_ + h^+^ → O_2(g)_), further increasing *τ*^2^,^3^,^4^,^5^. Very recently, Nahm *et al.* suggested, on the basis of their first-principles electronic structure calculations, that the light-induced bistability of substitutional hydrogen at oxygen sites could be the origin of PPC in ZnO^27^.

According to the previous discussion, the recombination of photo-excited carriers in the ZnO/Ag nanograting may take several routes and *τ* can be estimated by^3^


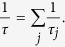


Sub-bandgap-energy light was used in our SPV measurements; hence, a fast band-to-band recombination with characteristics times in the nanosecond range cannot be included among the *τ*_*j*_ values in the aforementioned equation. The relaxation process in the sub-bandgap SPV could be very long due to very small thermal cross-sections (and hence capture coefficients) of the surface states^24^. In Fig. 6, the symbols represent measured SPV data and the dashed lines are the fitting curves according to the following equation: 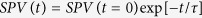. The estimated *τ* values are presented in Table 1. The *τ* values for the nanograting under TM-mode light and the flat sample (≥10^3^ sec) are similar; however, the *τ* value for the nanograting under TE-mode light (<10^3^ sec) is smaller than the others. Retamal *et al.* reported that the *τ* values related to the bulk recombination and surface oxygen desorption were 2.0 × 10^2^ sec. and 1.3 × 10^3^ sec respectively^5^. The *τ* value of our nanograting for the TM- (TE-) mode light is comparable to their surface (bulk) recombination time. The polarization dependence of *τ* for the nanogratings is very interesting, although an identical sample was used for both TM- and TE-mode illumination experiments.

SPV originates from the photo-generated excess carrier density near the surface. In particular, the holes created within the minority carrier diffusion length from the surface and recombination at the trap states mainly determine the SPV behaviors of the *n*-type ZnO layer^26^. As shown in Fig. 5, the SPP excitation significantly modifies the EM field patterns in the ZnO layers. The nanograting under TM-mode light and the flat sample have larger EM fields near the ZnO/Ag interface than the nanograting under TE-mode light. Such field maps directly affect the spatial distribution of the photo-excited carriers and the resulting recombination. As a result, the former two cases show somewhat similar *τ* values compared with the latter case (Table 1).

SPV of ZnO/Ag 1D nanogratings, as measured by KPFM, exhibited strong dependence on the wavelength and polarization of incident light. The dispersion relation of SPPs at the ZnO/Ag interface and optical simulations suggested that the SPP excitation caused this dependence. SPV became larger when SPP produced more charge carriers. SPV exhibited very slow relaxation because recombination at the trap states affected the quantity of excess charge carriers. The interaction between surface plasmons and charge carriers directly affected the creation and recombination processes of the charge carriers. Thus, SPV, originating from the photo-excitation of carriers and subsequent redistribution of net charges, helps us understand the plasmonic effects in metal/semiconductor hybrid structures.

## Methods

### Sample Fabrication

First, 1D grating patterns in a stamp consisting of polydimethylsiloxane (PDMS) were transferred to the UV-curable agent, trimethylolpropane propoxylate triacrylate (TPT), with the photoinitiator, 2-hydroxy-2-methylpropiophrone. ZnO/Ag thin films were grown by RF magnetron sputtering on the polymer patterns and also on planar Si substrates for comparison. The growth was performed at room temperature to avoid thermal damage to the polymer patterns.

### KPFM measurements

**Figure 2:**
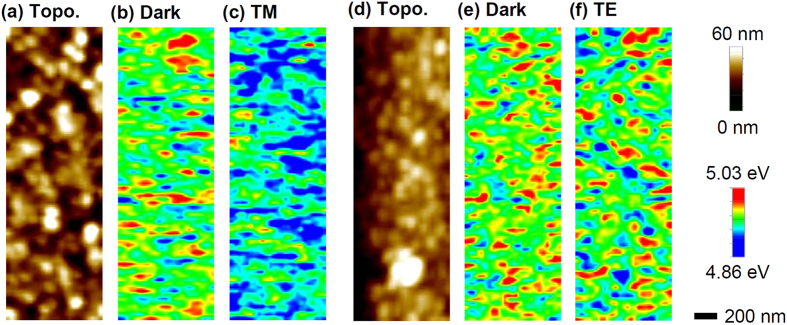
(**a**) Atomic force microscopy (AFM) topography image of the region on the nanograting surface used for the TM-mode SPV measurements. (**b**) *W*_*S*_ map without illumination and (**c**) *W*_*S*_ map under illumination by a TM-mode green light (wavelength: 532 nm) of the region in (**a**). (**d**) AFM topography image of the region used for the TE-mode measurements. (**e**) *W*_*S*_ map without illumination and (**f**) *W*_*S*_ map under illumination by a TE-mode green light of the region in (**d**).

The KPFM measurements were conducted using an atomic force microscopy system (XE-100, Park Systems Co.) inside a glove box. Conductive Pt-coated Si cantilevers (NSG10/Pt, resonance frequency: ~240 kHz, NT-MDT) were used for both work function (*W*_*S*_) and topography measurements. Immediately after each measurement, the work function of the tip was calibrated with a highly ordered pyrolytic graphite (HOPG, SPI Supplies) reference sample. After the glove box was purged with N_2_ for at least 3 h, the measurements were performed while maintaining a flow of N_2_ gas. Prior to the measurements, samples were stored in the dark for at least one day, to exclude the possible influence of persistent photocurrent. To estimate SPV, we measured the topography and *W*_*S*_ from a certain area on the sample surface in dark. And then, we measured the topography and *W*_*S*_ without moving the tip position with respect to the sample surface under illumination. We found the identical region from the scanned images using relative coordinates from a specific topographic feature on the sample surface. Figure 2a,d show the topographic images of the region used for the SPV measurements under TM- and TE-mode light illumination, respectively. The SPV values were obtained from each pixel of the *W*_*S*_ maps.

## Additional Information

**How to cite this article**: Gwon, M. *et al.* Plasmon-Enhanced Surface Photovoltage of ZnO/Ag Nanogratings. *Sci. Rep.* **5**, 16727; doi: 10.1038/srep16727 (2015).

## Supplementary Material

Supplementary Information

## Figures and Tables

**Figure 1:**
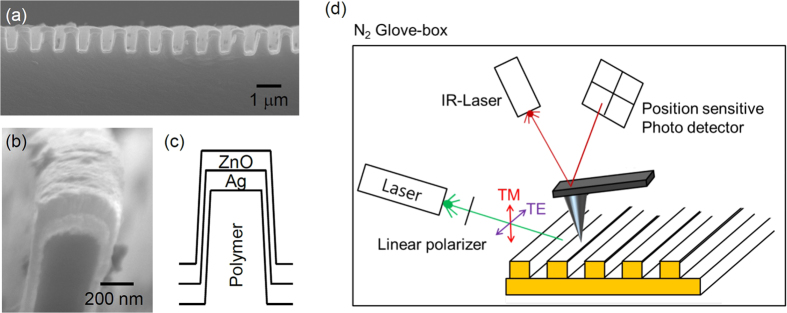
(**a**) Low- and (**b**) high-magnification cross-sectional scanning electron microscope images and (**c**) a schematic of a ZnO/Ag grating structure. (**d**) Schematic of the KPFM measurement setup in the glove box.

**Figure 3:**
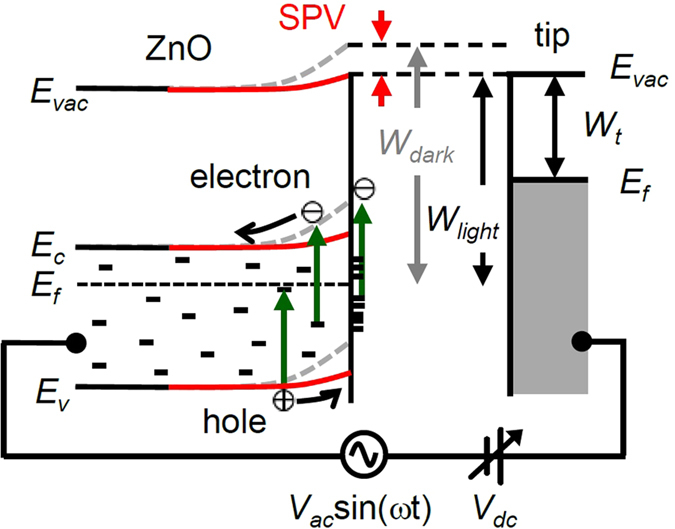
Schematic band diagram that explains the principle of SPV measurements of the ZnO surface using KPFM. Short bars represent trap states in the bulk and at the surface.* E*_*vac*_, *E*_*c*_, *E*_*f*_, and *E*_*v*_ indicate the vacuum level, the conduction band minimum, the Fermi level, and the valence band maximum. *W*_*dark*_, *W*_*light*_, and *W*_*t*_ correspond to the *W*_*S*_ in the dark, the *W*_*S*_ under illumination, and the work function of the tip. An AC bias voltage, *V*_*ac*_sin(*ωt*), and a DC bias voltage, *V*_*dc*_, are applied to the tip. The red solid lines (the gray dashed lines) describe the sample under illumination (in the dark). The lack of difference in *E*_*vac*_ at both the sample surface and the tip indicates that the electrostatic force between the tip and sample is nullified by *V*_*dc*_. In such a case, *V*_*dc*_ is equal to (*W*_*t*_ − *W*_*S*_) and the calibration of *W*_*t*_ using a proper reference (highly ordered pyrolytic carbon, in our experiments) allows us to estimate *W*_*S*_.

**Figure 4:**
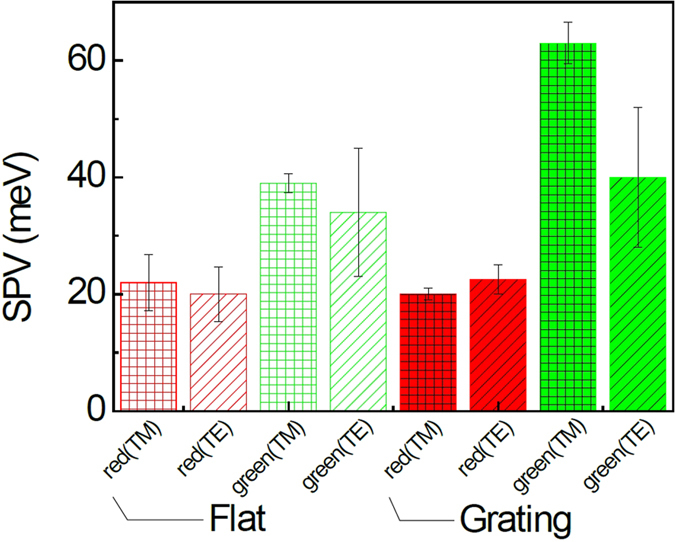
SPV of the ZnO/Ag flat thin film and nanograting samples under illumination with red and green light with TM- and TE-mode polarizations.

**Figure 5:**
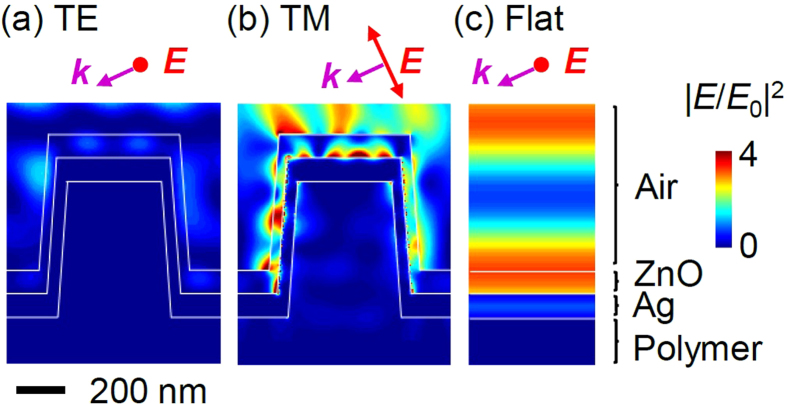
The electric field intensity distributions in the ZnO/Ag nanograting under. (**a**) TE-mode and (**b**) TM-mode green light (wavelength: 532 nm) illumination, and (**c**) the distribution in a flat ZnO/Ag/polymer/Si sample obtained by FDTD simulations. The electric field direction is parallel to the paper plane in (**c**). *E*_0_ indicates the magnitude of the electric field of the incident light. The wave vector (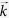) and polarization direction (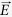) of the incident plane waves are indicated by purple and red arrows, respectively.

**Figure 6:**
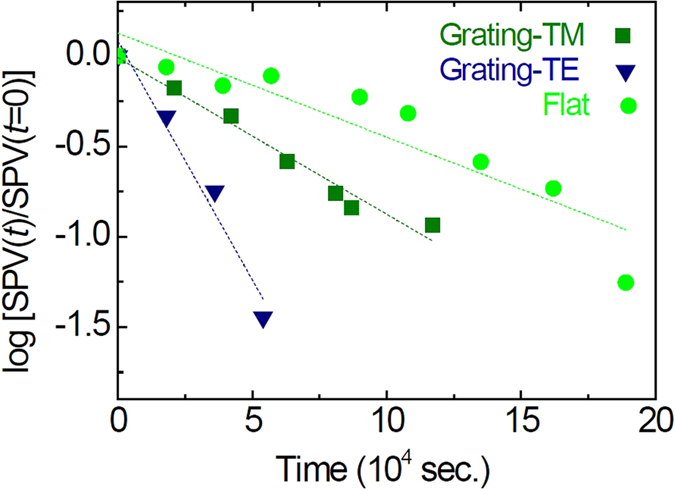
Normalized SPV of ZnO/Ag nanograting and a flat ZnO/Ag sample under green illumination in TM and TE modes, as a function of time. Dashed lines are exponential decay fitting curves.

**Table 1:** SPP decay time constant, *τ*, of grating and flat samples.

Sample	*τ* (10^3^ sec.)
Grating (TM mode)	1.2
Grating (TE mode)	0.38
Flat	1.7
