# Supplementary material for: Plasmon-Enhanced Surface Photovoltage of ZnO/Ag Nanogratings
Source: Sci Rep. 2015 Nov 16;5:16727. doi: 10.1038/srep16727 (PMC4645158; doi:10.1038/srep16727)
Supplement: Supplementary Information [file srep16727-s1.pdf]

Supplementary Information for

Plasmon-Enhanced Surface Photovoltage of ZnO/Ag Nanogratings

Minji Gwon,[1] Ahrum Sohn,[2] Yunae Cho,[3] Soo-Hyon Phark,[2,3] Jieun Ko,[4] Youn Sang Kim,[4] and Dong-Wook Kim[1,*]

[1] Department of Physics, Ewha Womans University, Seoul 120750, Korea

[2] Center for Correlated Electron Systems, Institute for Basic Science (IBS), Seoul 151-747, Korea

[3] Department of Physics and Astronomy, Seoul National University, Seoul 151-742, Korea

[4] Program in Nano Science and Technology, Graduate School of Convergence Science and Technology, Seoul National University, Seoul 151-742, Korea

* Correspondence and requests for materials should be addressed to D.K. (dwkim@ewha.ac.kr)

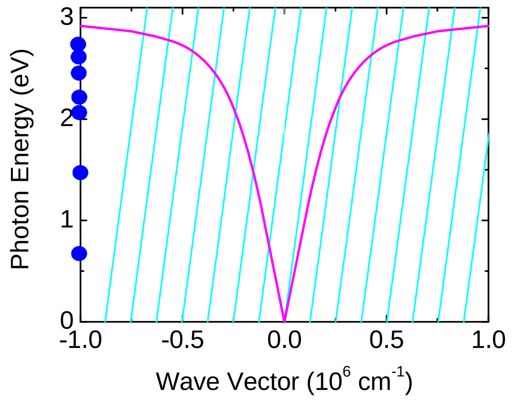

Figure S1: Dispersion relation of surface plasmon polariton (SPP) at the ZnO/Ag nanograting (color: magenta). Linear lines (color: cyan) correspond to the light lines with incident angle of 65°. Blue dots indicate the SPP energy that can couple incoming photons.

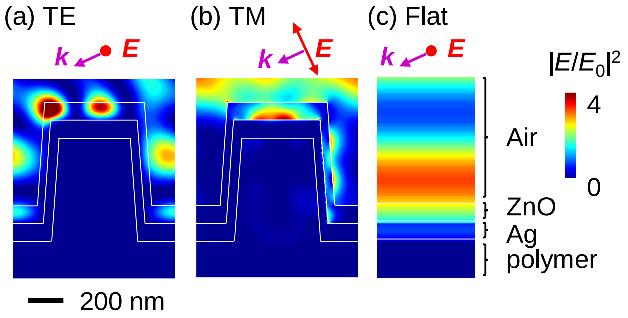

Figure S2: Electric field intensity distributions in the ZnO/Ag nanograting under illumination by (a) TE- and (b) TM-mode red light (wavelength: 635 nm). The field intensity distribution in a flat sample is compared in (c). The wave vector ($\vec{k}$) and polarization direction ($\vec{E}$) of the incident plane waves are indicated by purple and red arrows, respectively.
